# Multi-center disease-specific management system: toward standardized and harmonized clinical pathways in integrated care networks

**DOI:** 10.3389/fpubh.2026.1764254

**Published:** 2026-05-07

**Authors:** Xiang Liu, Rui Zhang, Tao Zheng, Ran Liu, Siyuan Zhang, Hongsen Zhao, Jing Dong, Siqi Chen, Jia Xu, Rui Shi

**Affiliations:** 1 Department of IT & Data Management, West China Hospital, Sichuan University, Chengdu, China; 2 Laboratory of Medical Artificial Intelligence, West China Hospital, Sichuan University, Chengdu, China; 3 Department of Orthopedics, West China Hospital, Sichuan University, Chengdu, China

**Keywords:** data governance, digital health platform, disease-specific clinical pathways, homogeneous disease management, medical alliance, multi-center, multidisciplinary collaboration

## Abstract

**Introduction:**

West China Hospital of Sichuan University leads the largest regional medical alliance in Western China, coordinating complex referrals and continuity of care. However, variability in clinical practices and the absence of a unified digital infrastructure have limited standardized disease-specific management across member institutions. This study aimed to develop and implement a standardized digital platform to support interoperable disease-specific management within the medical alliance.

**Methods:**

A total of 293 patients were enrolled in the early implementation phase. The system demonstrated feasibility in multicenter data integration, standardized pathway execution, and privacy-compliant cross-institutional data sharing. Preliminary deployment confirmed stable interoperability across participating institutions.

**Results:**

The results indicate that perceptions of increasing extreme weather are widespread among older adults in Austria, pointing to a broadly shared awareness of climate-related change. At the same time, perceptions are socially and spatially differentiated. Environmental awareness emerges as the strongest correlate of perceived increases, underscoring the importance of cognitive and informational factors in shaping climate-related interpretations. Urban residence is associated with stronger perceptions compared to town and rural living, suggesting the relevance of geographic context and everyday exposure. Higher education and financial hardship are also positively associated with perceived increases. In contrast, subjective health and loneliness show no significant associations.

**Discussion:**

The W-SDM System provides a scalable and standardized digital framework for pathway-based disease management within a medical alliance. This early-stage implementation establishes a foundation for future evaluation of clinical effectiveness, workflow optimization, and long-term outcome assessment.

## 1 Introduction

Medical alliances (i.e., Integrated Care Networks) represent a key organizational model in China for advancing tiered diagnosis and treatment and enhancing continuity of care. These alliances aim to reduce inter-institutional service barriers, facilitate resource integration and vertical collaboration, and disseminate high-quality medical resources to primary care settings. The ultimate goal is to establish patient-centered care pathways encompassing pre-diagnosis, treatment, and follow-up (1). Such mechanisms contribute to optimizing the allocation of healthcare resources, alleviating challenges associated with “difficult and expensive access to care,” and promoting the standardization and efficiency of medical services. The World Health Organization (WHO) defines integrated health care as the provision of continuous and coordinated medical services across different levels and settings of care, covering the entire life course (2). In China, medical alliances serve as the institutional embodiment of this concept, particularly in managing chronic diseases and multimorbidity, where seamless coordination from initial consultation and referral to rehabilitation and follow-up is emphasized (1, 3–5).

Amid ongoing digital transformation, innovations in digital health services are recognized as drivers of regional healthcare development and tools for optimizing resource allocation (6). Although digital health technologies can improve health outcomes and accessibility, international studies indicate that they may exacerbate inequities across regions and populations (7). Leveraging digital technologies to enhance inter-institutional collaboration and promote homogeneous services has been validated in multiple models. For example, a telemedicine-based, village doctor-led integrated care model for atrial fibrillation (AF) significantly improved adherence to the ABC pathway and enhanced cardiovascular outcomes (8). Internationally, the experience of Kaiser Permanente in the United States illustrates how a highly unified electronic health record (EHR) platform, HealthConnect, can facilitate clinical integration across institutions, support shared decision-making, and implement standardized care pathways. This model has substantially improved clinical outcomes and operational efficiency by integrating decision support and standardized care pathways throughout the continuum of care (9).

Since 2013, China has accelerated the establishment of medical alliances (MAs). By the end of 2023, more than 18,000 MAs had been established nationwide, supported by over 50 national policy documents aimed at promoting their development (10). As the largest medical center in western China, West China Hospital of Sichuan University launched the West China Hospital Medical Alliance, comprising 18 partner hospitals across Southwest China. This alliance encompasses key functions such as specialty consortium development and two-way referral services. By June 2025, the 18 member institutions had carried out two-way referral services. In practice, limitations in information technology infrastructure have emerged as a key bottleneck, constraining efficiency and collaborative capacity. Member institutions exhibit substantial variability in digital maturity, with diverse system types and inconsistent data standards. Primary and secondary hospitals, in particular, often face financial and technical constraints that hinder the provision of essential services such as patient registration, referral, and examination result sharing (11). The absence of a unified information system hampers service delivery and limits the potential benefits of MAs in care coordination, resource sharing, and quality control. A survey of 640 healthcare professionals within the West China Hospital MA further revealed considerable gaps in knowledge, attitudes, and practices (KAP) related to alliance-based care. Satisfaction with the existing information system was the lowest among the assessed domains. Most respondents identified the lack of a unified IT framework and efficient data-sharing mechanisms as the main barrier to effective collaborative diagnosis and treatment within the alliance (12).

This study builds upon the central coordinating role of West China Hospital of Sichuan University within its regional medical alliance. We developed the West China Specialty Disease Management System (W-SDM System), a standardized and interoperable digital platform designed to support pathway-centered disease-specific management across member institutions. The platform enables structured dissemination of clinical pathways, role-based collaboration through mobile and PC applications, real-time patient enrollment and data reporting, and secure integration with the hospital’s central big data infrastructure. This article presents the development and early deployment of the system, describing its architecture, operational framework, and initial application within the regional alliance. By establishing a unified digital infrastructure, the W-SDM System provides a foundation for standardized disease management and sustained regional healthcare collaboration.

## 2 Materials and methods

The W-SDM System was developed as a digital infrastructure to operationalize disease-specific clinical pathways within a regional medical alliance led by West China Hospital of Sichuan University. The platform connects 11 affiliated hospitals and supports role-based access for frontline physicians, central hospital clinicians, and disease-specific expert advisors. The system enables structured pathway implementation, standardized data collection and structuring, and centralized data integration to support cross-institutional coordination and quality monitoring. The platform provides a digital infrastructure to support the implementation of standardized clinical pathways across institutions and facilitate coordinated disease management within the alliance.

### 2.1 System functional design

Based on the practical requirements of disease-specific management within a regional medical alliance and aiming to overcome the spatial limitations of traditional healthcare delivery, the W-SDM System was designed as a role-based, multi-terminal collaborative platform. The system comprises three coordinated modules: a Mobile Application for alliance physicians, a Mobile Terminal for central hospital physicians, and a PC Portal for principal investigators, together forming a pathway-centered framework for cross-institutional disease-specific management. The Mobile Application enables alliance physicians to enroll patients, perform disease classification and TNM staging, longitudinally document clinical progress, and compare actual care against standardized treatment pathways. Real-time progress monitoring, quality-control alerts, and structured messaging support adherence to pathway standards. The Mobile Terminal allows central hospital physicians to compare structured case data submitted by alliance institutions with clinical pathways, providing pathway optimization recommendations, teleconsultation, and referral coordination. The PC Portal supports experts and principal investigators in multicenter data monitoring, pathway quality evaluation, and dynamic pathway optimization, generating structured feedback to continuously refine alliance-wide clinical practice.

System interoperability is achieved through four structured mechanisms: (1) the mobile interface connects to the Internet Hospital Platform via an integrated service gateway, enabling visit-tag generation and online service activation; (2) a secure dedicated transmission line links the system to the Big Data Center and the Image Cloud Platform, supporting standardized exchange of disease-specific clinical and imaging data; (3) a Unified Interface Service integrates the system with central hospital applications, including the Remote Consultation and Referral Platform and the Unified API Service Platform, ensuring seamless cross-system data flow; and (4) standardized clinical pathway data structures facilitate real-time progress tracking, quality-control processing, and coordinated cross-institutional supervision.

**Figure 1:**
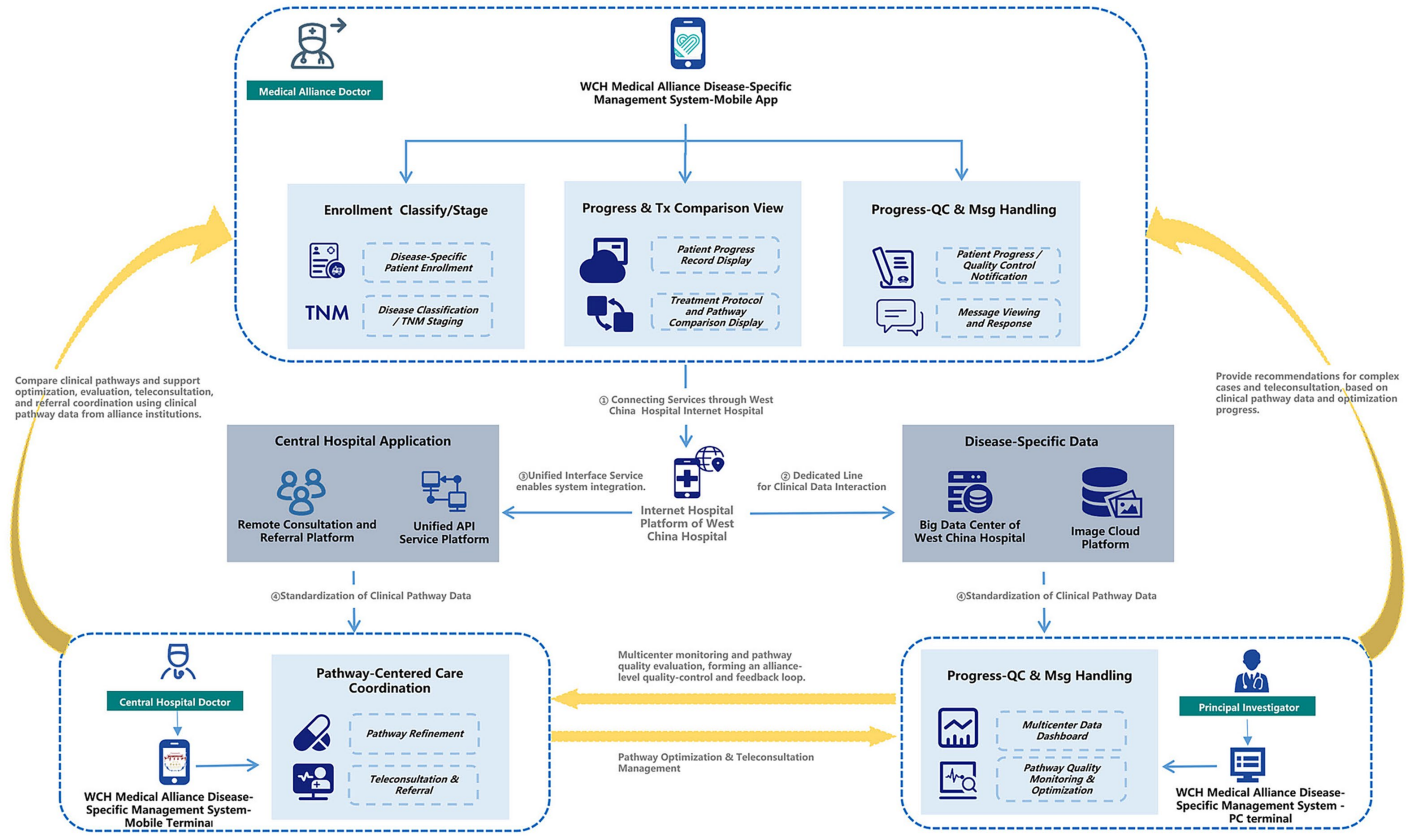
Inter-system interaction, data flow, and workflow execution in the West China Medical Alliance Disease-Specific Management System.

Through this architecture, the W-SDM System establishes a closed-loop, pathway-centered collaborative model: alliance physicians use the mobile application to enroll patients, document TNM staging, and record pathway execution; central hospital physicians compare structured case data against clinical pathways to provide optimization recommendations, teleconsultation, and referral coordination; and disease experts and principal investigators use the PC portal to monitor multicenter data, evaluate pathway quality, and perform dynamic optimization. This continuous feedback loop enables alliance-wide quality control and iterative refinement of clinical practice (Figure 1).

### 2.2 Project objectives and development strategy

In this study, the development of a digital platform for disease-specific management within the medical alliance encountered multiple challenges, including the coordination of multidisciplinary teams, cross-institutional collaboration among hospital information centers, heterogeneity in digital infrastructures, and considerable variability in disease-specific team requirements. Expectations regarding platform functionality, data models, and workflow specifications differed substantially across institutions. In addition, the initial requirements were frequently underspecified and subject to dynamic revisions during implementation. Compounding these challenges, the entire process—from requirements elicitation to system deployment—was required to be completed within 90 working days, which imposed stringent demands on both requirements engineering and development strategy.

**Figure 2:**
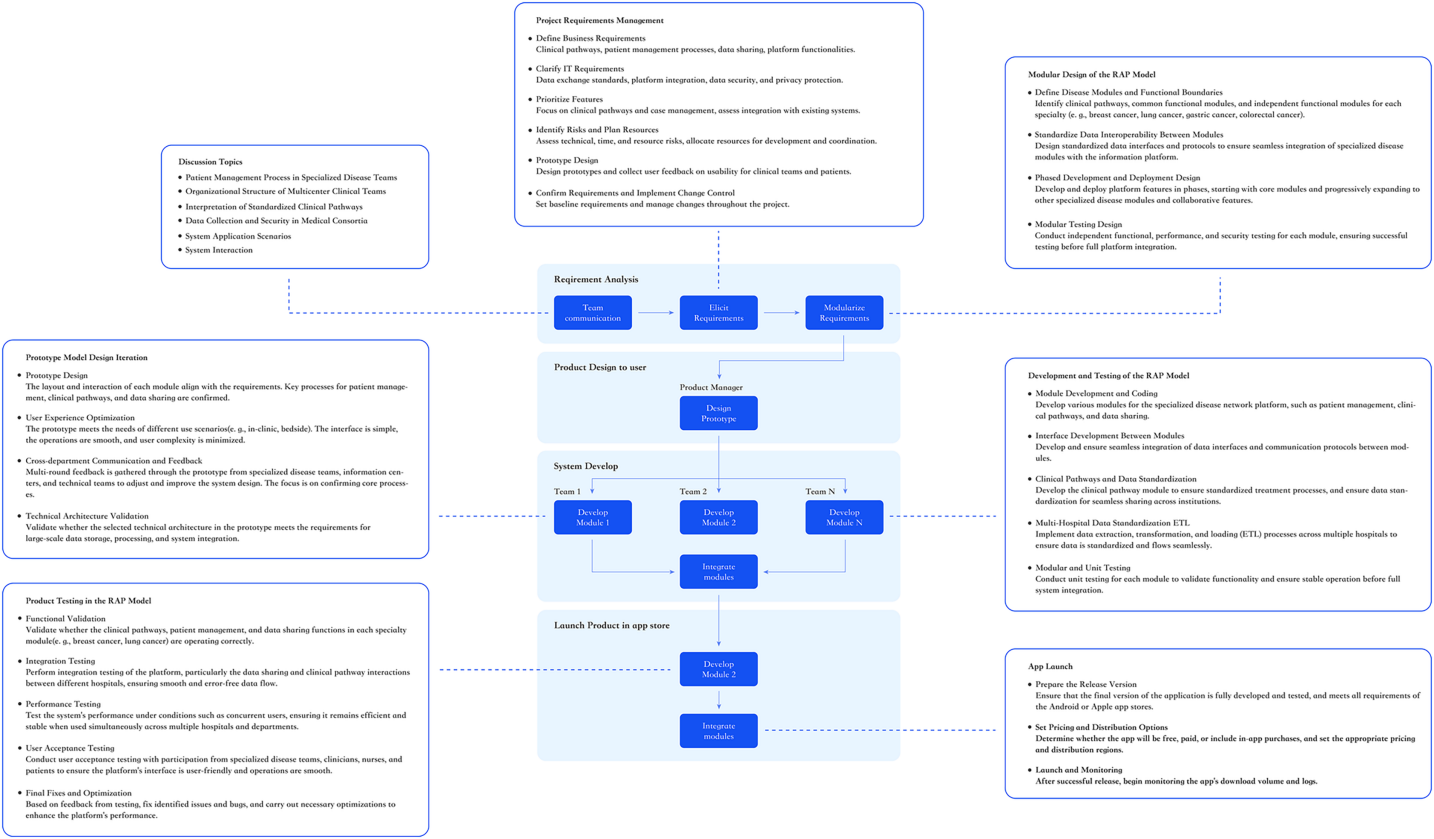
Hybrid development strategy, prototyping model, and RAD model in medical system design and implementation: (1) Interviews and discussions: disease-specific management, clinical teams, pathway interpretation, data security, and system design. (2) Project requirements management: define business and IT requirements, prioritize functionalities, assess risks, prototype design, and change control. (3) Modular design of the RAD model: clarify requirements, optimize user experience, feedback and communication across departments, and validate technical architecture. (4) Prototype model design iteration: adjust requirements bias, optimize user experience, cross-department feedback, and technical architecture validation to ensure system alignment with clinical needs. (5) Development and testing of the RAD model: module development, interface development, data standardization, ETL processes, and unit test design. (6) Product testing in the RAD model: functional validation, integration testing, performance testing, user acceptance testing, and issue resolution to ensure system stability. (7) App launch: prepare the release version, set pricing and distribution strategy, monitor downloads, user behavior, and system logs post-launch.

To address these challenges, we adopted a hybrid development strategy that combined the Prototyping Model and the Rapid Application Development (RAD) Model (13, 14). The Prototyping Model enabled the rapid construction of functional prototypes to capture and refine early-stage requirements, thereby aligning stakeholders on system objectives and functionalities. In parallel, the RAD Model emphasized modularization and concurrent development, ensuring responsiveness to evolving requirements while maintaining development quality and shortening the project timeline (15). The integration of these two approaches was particularly suited to multi-hospital, multi-team, and cross-system collaboration, facilitating consensus among diverse hospitals and disease-specific teams under a unified platform objective, and thereby ensuring streamlined project implementation (Figure 2).

During the requirements management phase, IT requirements and functional priorities were first defined based on representative clinical scenarios. The scope of first-phase deliverables was delineated, and a baseline functional checklist was established (Table 1). Multiple rounds of discussions and prototype iterations were subsequently conducted with clinicians and multicenter IT teams to ensure that key processes were aligned with clinical workflows. This also allowed alliance hospitals to quickly gain an understanding of system objectives and prepare for local system integration. A baseline requirements specification was finalized upon consensus among stakeholders.

**Table 1:** Development modules and key software engineering activities of the West China medical alliance disease-specific management system.

Module	Function/feature	Description
System configuration	Import of user/role/alliance organization information	Maintenance of user information; configurable roles and permissions
	Operation logs/error logs recording	Backend system monitoring and bug information collection
	Specialty management: display specialty list; create, edit, delete specialty forms	Unified specialty templates for add/delete operations to ensure maximum compatibility with current known specialties
	Role binding	A single user can possess multiple role-based permissions
	Specialty pathway form configuration – maintenance of specialty treatment phases	Configurable specialty pathway items for lung cancer, gastric cancer, breast cancer, and colorectal cancer
	Specialty pathway form configuration – maintenance of diagnostic and therapeutic item dictionary for each phase	Configurable dictionaries for pathway items for lung cancer, gastric cancer, breast cancer, and colorectal cancer
	Specialty pathway form configuration – maintenance of quality control scoring standards dictionary	Attribute management for scoring points at each node of the specialty pathway, each node in the specialty pathway project is configurable, and common nodes are reusable.
Specialty patient management (mobile terminal)	Dashboard: specialty patient registration, message module, indicator board	Entry point for each application module
	Patient admission: id card scanning, upload of informed consent photos, basic information filling	Obtain patient basic data via ID card scan; automatic ID number recognition and correction
	Quality control messages: message list, notifications, list query, message replies	
	Data details: patient list, stage-wise data display, comparison between standard and actual pathways, evaluation viewing and replies	
	Internet hospital integration: push admitted patients, identity document recognition (doctor side); message viewing (patient side)	Internet hospital platform acts as the app entry point
Specialty patient management (PC terminal)	Specialty physician (West China) admission via HIS: West China specialty physicians admit patients via HIS (Hospital information system)	
	Patient list: query admitted patient list, quality control message alerts	
	Data details: stage-wise data display; standard vs. Actual pathway comparison (pathway execution comparison); evaluation view and reply; imaging review	Structured visualization of predefined clinical pathway nodes is implemented, with real-time mapping of actual clinical actions to their corresponding nodes (e.g., verification of whether pathway-specific medications and procedural orders have been executed).
Clinical pathway quality control (PC terminal)	Automated TNM staging validation and alerts	For enrolled oncology patients, mandatory reminders are triggered when TNM staging information is missing. Cases without documented staging are automatically prioritized in the patient list interface, and quality control personnel are notified to issue follow-up reminders to responsible clinicians.
	Patient list: query admitted patient list, message reply reminders	
	Data details: stage-wise data display, standard vs. actual pathway comparison, annotations and evaluations	
Patient terminal	View messages: access detailed notifications regarding specialty care	
Indicator dashboard (PC terminal)	Specialty management indicator dashboard	Indicator data visualization by disease type; patient count by hospital; hospital-specific patient count for each disease type; team size; proportions of histological subtypes and TNM stages in lung cancer
	Management indicator dashboard	Indicator display by hospital: proportion of patients per specialty, number of specialty physicians, patient admission trends per specialty
Indicator dashboard (mobile terminal)	Consistent with the pc-side indicators	
Data acquisition and governance	Sync admitted patient information to the data center	This project aims to synchronize the basic information of admitted patients to the data center, where the central hospital assigns standard form templates and ETL scripts.
	Sync admitted patient information to alliance hospital clinical data gateway	This project involves syncing the admission patient data to the clinical data front-end of medical consortium hospitals, ensuring timely access and sharing of patient data within the hospital, supporting real-time diagnosis and cross-hospital collaboration.
	Alliance hospitals extract and upload visit data for admitted patients	This project requires medical consortium hospitals to develop and process the visit data of admitted patients and upload it to the designated database or platform for further processing and monitoring within the system, ensuring data integrity and accuracy.
	Data quality control scripts	The central hospital uniformly distributes data detection scripts for automated testing of the integrity, consistency, and accuracy of admission patient data, ensuring no data loss or errors during uploading and processing, thus supporting high-quality data management.
	Integrate imaging interface to pull admitted patients’ imaging data	Imaging files are retrieved from alliance hospitals based on admitted patients’ examination information

During the development phase, the RAD Model was applied to facilitate modular design and parallel development. Functional modules were independently developed by different centers, with unit and module testing conducted concurrently. Although this approach entailed an increased overall testing workload, it significantly improved system stability and security and contributed to a substantial reduction in the project timeline. Subsequent integration testing, performance testing, and user acceptance testing ensured deployment quality. Post-deployment, system optimization was continuously conducted based on user download and activity logs to further enhance system performance and user experience.

This hybrid strategy—combining rapid requirement alignment via the Prototyping Model with efficient development execution via the RAD Model—enabled on-schedule deployment of the platform in a multi-institutional, multi-system, and heterogeneous environment. It also supported disease team–specific customization, ensuring the platform’s usability and scalability in real-world clinical practice.

### 2.3 Multicenter data interaction architecture within the medical alliance

**Figure 3:**
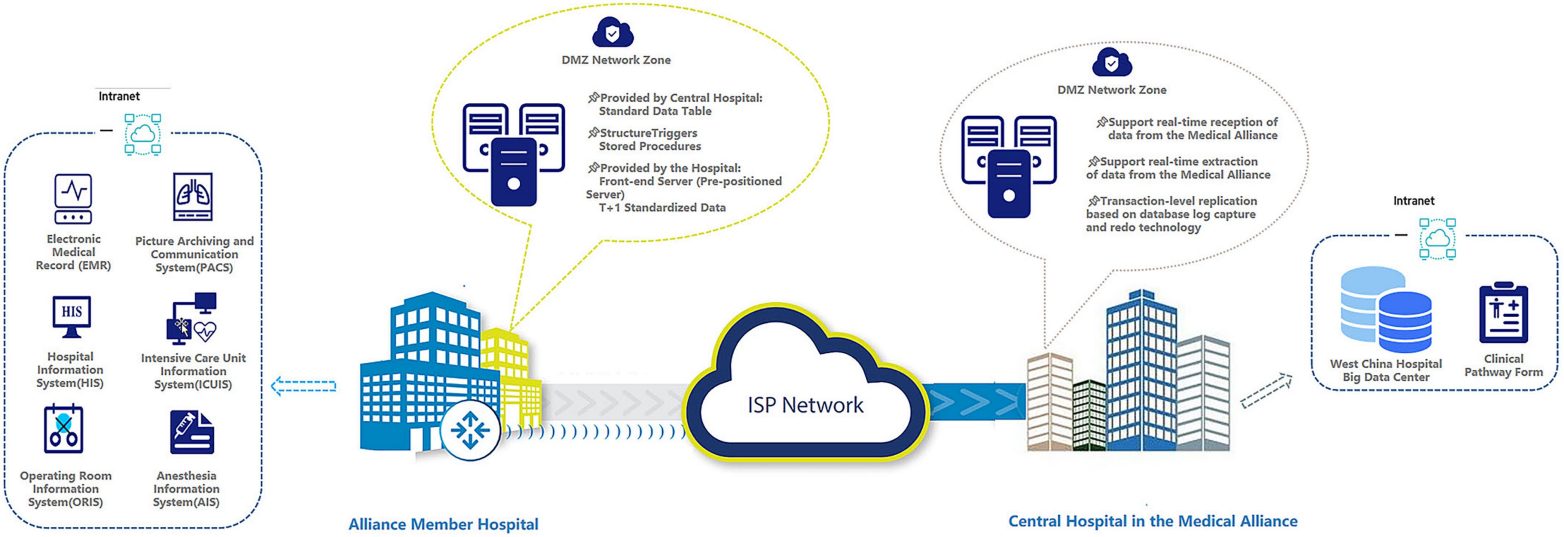
Multi-center data sharing within the medical alliance.

A unified data interconnection framework is essential for collaborative management and treatment standardization within a disease-specific medical alliance. To this end, we designed a multicenter data interaction architecture linking alliance member hospitals with the central hospital (e.g., West China Hospital), enabling secure, efficient, and standardized data sharing across the network. The architecture is highly scalable and replicable, adopting a hybrid communication model that combines real-time data transmission with T + 1 structured data synchronization, thereby balancing timeliness and accuracy for cross-hospital clinical collaboration (Figure 3). Both the central and member hospitals deploy DMZ isolation zones as security buffers and configure front-end data servers. The central hospital uniformly provisions member hospital servers with standardized data interface forms, data verification stored procedures, and table-building scripts for 71 standardized data collection tables. This ensures consistency in data structure, content, and quality. Data are collected from multiple core clinical information systems, including the Electronic Medical Record (EMR), Hospital Information System (HIS), Picture Archiving and Communication System (PACS), Intensive Care Unit Information System (ICUIS), Operating Room Information System (ORIS), and Anesthesia Information System (AIS). Each member hospital collects data according to unified standards and synchronizes standardized data to the central hospital on a T + 1 (daily) basis.

For this study, a dedicated big-data communication line was implemented to support inter-hospital data exchange. This approach guarantees network security, stability, and efficiency (16), while also ensuring data privacy. Data exchange relies on ISP-based transmission channels, with primary link bandwidth ranging from 10 Mbps to 10 Gbps, supplemented by backup links such as ADSL and EFM to enhance disaster recovery. The central hospital’s front-end servers can receive and extract member hospital data in real time, supporting transaction-level replication using database log capture and redo mechanisms. All data are ultimately consolidated into the West China Hospital Big Data Center, providing robust support for dynamic optimization of clinical pathways and quality control in disease-specific management.

Data quality is the cornerstone of interoperability, as both clinical practice and research are increasingly dependent on reliable datasets. The utilization of Electronic Health Record (EHR) data has gained growing attention worldwide (17, 18). However, the establishment of unified data standards and quality control mechanisms remains one of the major challenges for medical alliance data centers (19, 20). Building on the clinical pathway management framework of West China Hospital and prior recommendations for the informatization of clinical pathways (21), we designed 71 standardized data collection forms for the medical alliance. These forms include 52 clinical diagnosis and treatment forms, 11 diagnostic imaging forms, and 8 laboratory testing forms.

Specifically, the 52 clinical forms were designed with reference to the standards of the national “Three Medical Supervision” platform (22); the 11 imaging forms were fully aligned with the Sichuan Provincial Health Commission’s Standards for Data Collection of Imaging Information Sharing and Retrieval (Trial); and the 8 laboratory testing forms strictly followed the Sichuan Provincial Health Commission’s Standards for Data Collection of Laboratory Information Sharing and Retrieval (Trial). Furthermore, all data quality requirements were benchmarked against the Sichuan Provincial Standards for Medical Institution Docking of Laboratory and Imaging Result Sharing and Retrieval (Trial) and the Sichuan Provincial Standards for Platform Docking of Laboratory and Imaging Result Sharing and Retrieval (Trial) (23).

**Figure 4:**
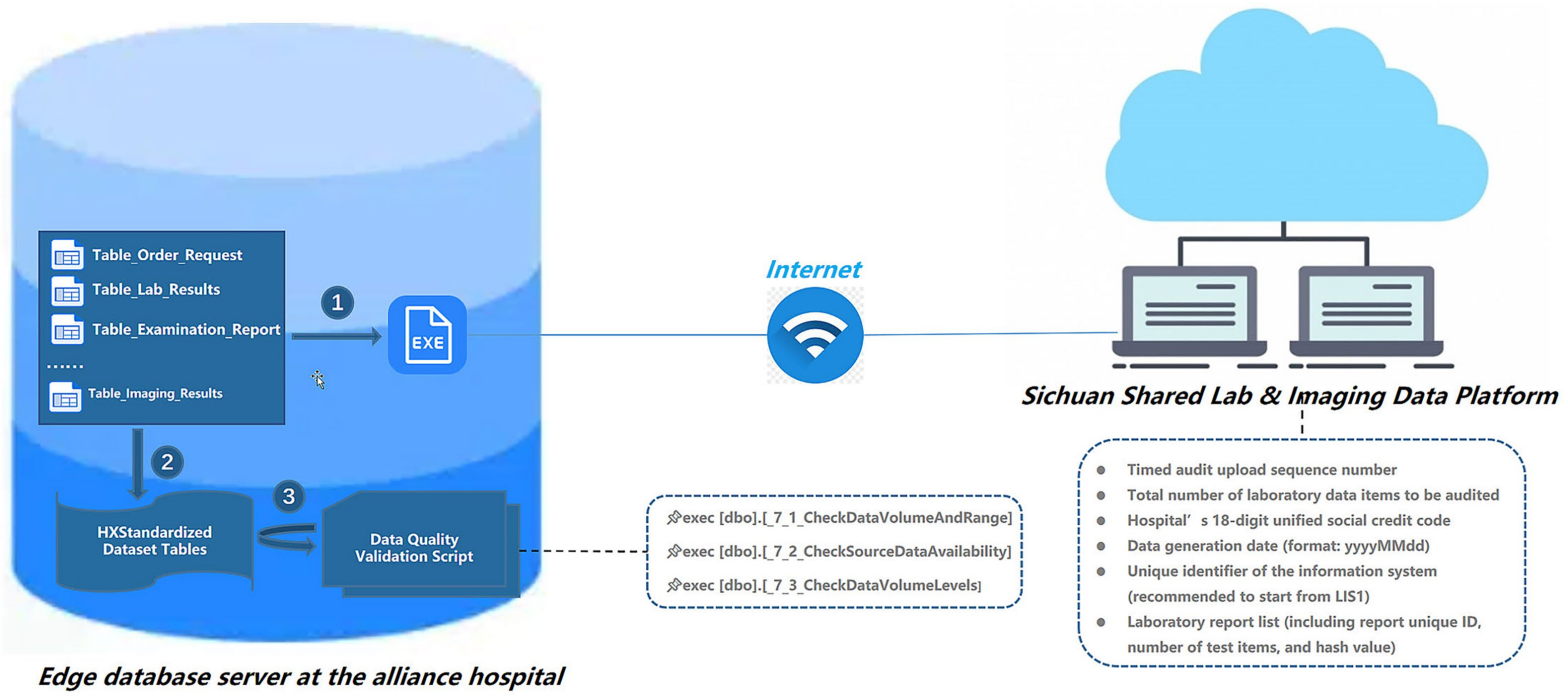
Data quality validation process: (1) Initial validation conducted through the provincial-level health data platform; (2) Data extraction into West China Medical Center standardized templates; (3) Final validation based on West China Medical Center data standards.

The data quality assurance workflow is illustrated in Figure 4. The first step involves ensuring the integrity and consistency of laboratory and imaging data. Hospitals periodically collect inspection and laboratory data in compliance with standardized specifications. The ID list of laboratory records is uploaded via a standard interface to the Sichuan Shared Lab & Imaging Data Platform, after which the corresponding structured datasets are automatically uploaded to the platform. The dataset includes unique identifiers, the total number of tests, timestamp of generation, and the hospital’s unified social credit code, thereby ensuring data integrity and tamper-proofing. Upon receipt, the platform automatically performs integrity verification and guarantees consistency between uploaded and source data. In addition, each upload is recorded with sequence numbers and log information, forming a complete audit trail. In the second step, laboratory and imaging data verified by the Sichuan Shared Lab & Imaging Data Platform are extracted into disease-specific forms published by West China Hospital. To facilitate this process, the central hospital provides a unified data quality validation script. Drawing on domestic and international practices, data quality is typically evaluated across multiple dimensions, including completeness, validity, consistency, security, timeliness, and interoperability (24). In this study, our validation framework was organized into four key categories: Data Integrity, Data Consistency, Cross-System Consistency, and Data Fusion Quality (see Supplementary Table)

The verification process included the following steps: (i) assessing the number of records in each table as well as the earliest and latest timestamps to understand data coverage and continuity; (ii) checking whether each table had been successfully imported from the corresponding operational systems (e.g., HIS, EMR, LIS) to evaluate the validity of system interfaces and ensure data completeness; (iii) performing a stratified evaluation of table-level data volume against predefined thresholds to promptly identify potential “pseudo-imports” or insufficient data; and (iv) conducting data consistency checks. In this study, seven data validation rules were designed, covering key aspects of patient encounters, medication, billing, hospitalization, and surgical procedures, thereby establishing a closed-loop framework for data quality assessment to ensure completeness, consistency, and reliability across multiple systems. Specifically, the total number of actual inpatient admissions was first determined to verify the completeness of baseline patient data, serving as a reference for all subsequent validations. Next, outpatient and inpatient billing data were cross-checked to confirm the integrity of the linkages among charge details, patient master index, and identity records. Patient identity consistency was further verified by matching ID numbers between the patient information table and inpatient records, followed by cross-validation of medication and surgical records to ensure internal consistency. Additionally, prescription data were examined to assess cross-system consistency, and inpatient records were reconciled across databases to verify the quality of data integration. These validation procedures provide a comprehensive approach to confirming the accuracy and reliability of patient data across heterogeneous systems.

### 2.4 User interaction design

#### 2.4.1 Structured clinical pathways

A pivotal component of the system is the structured clinical pathway (CP) form, designed to capture patients’ longitudinal encounters across institutions within the medical alliance. The form decomposes the clinical process along two dimensions—temporal stages (e.g., initial consultation, follow-up, treatment, surveillance) and clinical categories (e.g., screening, demographic and baseline information, diagnostic results, TNM staging, therapeutic interventions)—and explicitly defines the indicators to be collected at each node, their semantic specifications, and the corresponding structured data elements. The captured content integrates core EHR components (chief complaints, medical and family history), diagnostic outputs (imaging and laboratory reports), therapeutic orders (surgery, chemotherapy, targeted and immunotherapy), and molecular testing results, thereby ensuring traceability and persistent data capture throughout the entire pathway (Supplementary Table). Leveraging this structured form, the system supports automated conformance checking of real-world patient trajectories against standardized disease-specific pathways, enables visualization of deviations, and establishes a robust data foundation and toolset for pathway optimization, workflow refinement, and quality assurance.

#### 2.4.2 UI design and description

**Figure 5:**
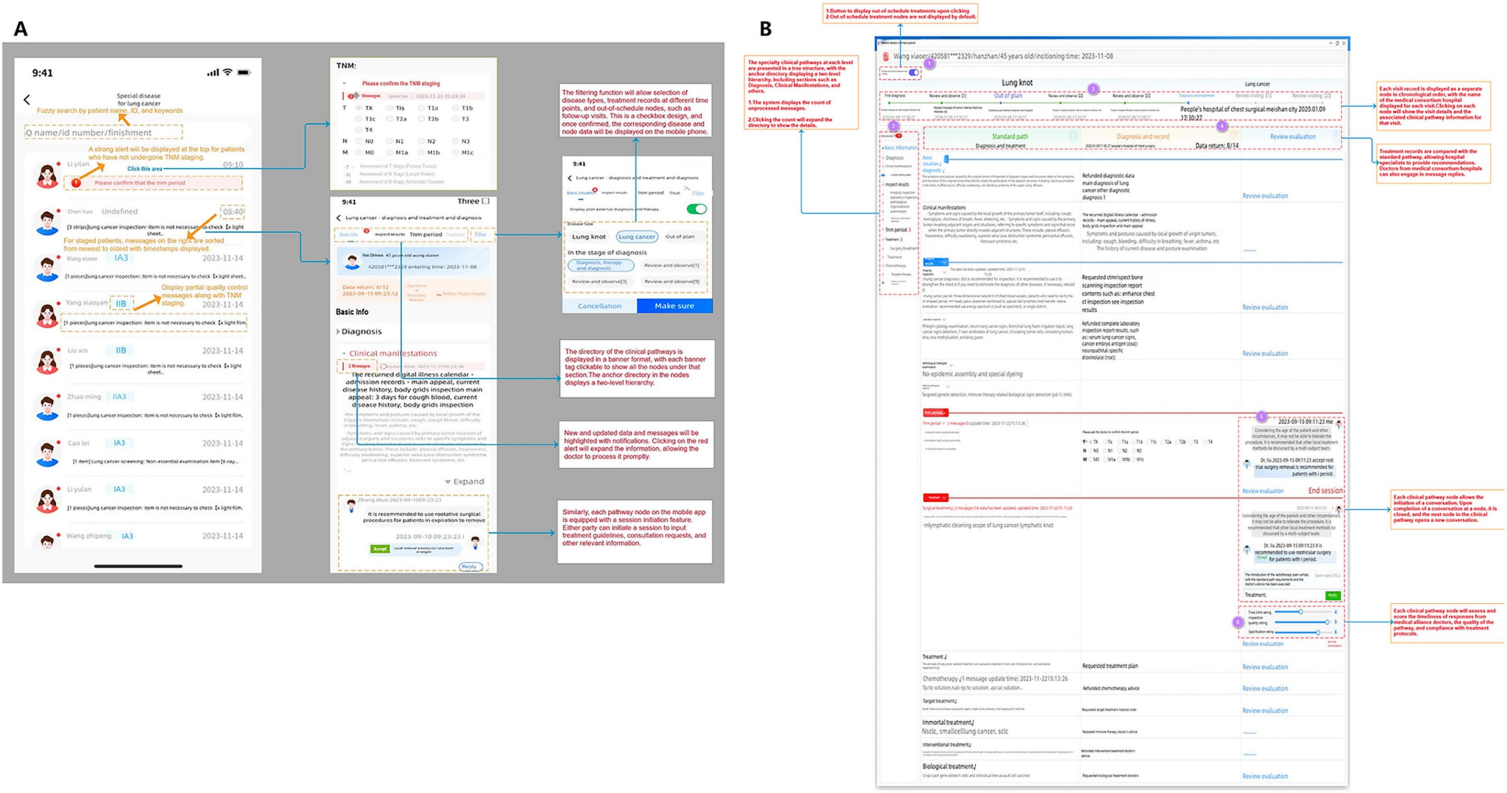
Structuralization of disease-specific pathways and user experience (UE) design: **(A)** Interaction design for patient management on mobile devices and disease-specific pathway management; **(B)** Interaction design for disease-specific pathway management tailored to PC in central hospitals.

The W-SDM system incorporates a clinician-oriented user interface (UI) design that conforms to the reading and operational habits of clinical staff. By adopting an intuitive and structured visual layout, it enhances information accessibility and interface usability, thereby improving workflow efficiency and supporting evidence-based decision-making (25, 26). Separate interfaces for patient management and form operations were optimized for both PC and mobile platforms (Figure 5), allowing clear presentation of patients’ clinical status across longitudinal encounters and disease-specific pathway stages. This dual-interface design enables precise clinical decision support while fostering team engagement and adherence throughout pathway execution (27–29).

The mobile interface Prototype (Figure 5A; a high-resolution version is provided in Supplementary Figure S1), primarily targeting frontline physicians, operationalizes standardized clinical pathways at the point of care. It integrates structured data visualization, decision-support alerts, and interactive communication functions within a concise, clinician-oriented UI. The system supports fuzzy search by patient name, ID number, or keywords for rapid record retrieval. Incomplete TNM staging triggers high-visibility alerts, with mandatory reminders to ensure timely data entry. For staged patients, TNM information is displayed with embedded quality-control prompts, all messages timestamped and ordered in reverse chronology for traceability. Pathway content is displayed via a two-level hierarchical banner, allowing rapid navigation from pathway phases to intervention nodes. Each node hosts structured data forms, which are directly compared against the standard pathway to generate quality-control prompts and guide compliance with documentation norms. A filtering module enables multi-condition queries by disease type, pathway stage, or unplanned events, while critical alerts link directly to detailed records. Each node also incorporates collaboration functions for initiating discussions, consultation requests, or shared reviews.

The PC interface Prototype (Figure 5B; a high-resolution version is provided in Supplementary Figure S2), designed for central-hospital experts, emphasizes centralized presentation of structured data and cross-institutional collaboration. The left panel provides a two-level hierarchical directory by pathway phase and node, highlighting missing staging records or quality-control anomalies. The main workspace organizes selected node data into modular columns—diagnoses, structured forms, and quality-control prompts—facilitating horizontal comparison of actual care against pathway standards. Updates are prominently highlighted, and the right-side conversation panel supports real-time inter-institutional consultation, allowing experts to adjust care plans or provide quality-control feedback in context. Alerts and updates are synchronized via backend transaction-level replication, while the filtering function leverages a unified data warehouse to support multi-condition, cross-institutional queries. Compared with the mobile interface, the PC interface de-emphasizes touch-based operations but strengthens batch case review, quality-control evaluation, and regional collaboration, serving as the central platform for expert-led pathway management and governance in the medical alliance.

To further demonstrate the operational mechanisms of key decision-support functions under real-world deployment, two Supplementary Figures derived from the officially deployed system are provided. Supplementary Figure S3 illustrates the high-visibility mandatory alert triggered for patients with incomplete TNM staging, reflecting the system’s enforcement strategy to ensure staging completeness and data integrity. Supplementary Figure S4 presents the clinical pathway deviation tracking interface, which visualizes deviations from standardized pathways and facilitates structured feedback and corrective actions. Together, these supplementary materials substantiate the W-SDM system’s capacity for real-time monitoring, deviation management, and closed-loop quality control within the medical alliance.

## 3 Results

### 3.1 Registration of disease-specific teams and patient enrollment within the medical alliance

This study involved 11 hospitals within the Sichuan University West China Hospital medical alliance, with the system initially implementing four disease-specific clinical pathways for lung cancer, breast cancer, gastric cancer, and colorectal cancer. In the lung cancer pathway, 148 physicians from 8 member hospitals participated, including 68 (45.9%) with associate senior or higher professional titles. The multidisciplinary team (MDT) comprised thoracic surgery, respiratory and critical care medicine, oncology, laboratory medicine, radiology, pathology, interventional medicine, and radiation oncology. For the breast cancer pathway, 117 physicians from 9 hospitals were enrolled, 55 (47.0%) of whom held associate senior or higher titles, representing breast surgery, radiology, pathology, oncology, ultrasound, and general surgery. The gastric cancer pathway included 113 physicians from 9 hospitals, with 53 (46.9%) at the associate senior level or above, covering gastroenterology, gastrointestinal surgery, hepatobiliary-pancreatic surgery, cardiothoracic surgery, pathology, oncology, and radiation oncology. In the colorectal cancer pathway, 95 physicians from 7 hospitals participated, including 46 (48.4%) with associate senior or higher titles, from gastrointestinal surgery, gastroenterology, hepatobiliary-pancreatic surgery, cardiothoracic surgery, oncology, and endoscopy centers. Across all pathways, broad multidisciplinary engagement and a high proportion of senior physicians underscored the medical alliance’s strong commitment to clinical pathway implementation. This level of participation ensures that patient care is guided by experienced multidisciplinary teams, thereby enhancing the quality, comprehensiveness, and standardization of oncologic management.

**Figure 6:**
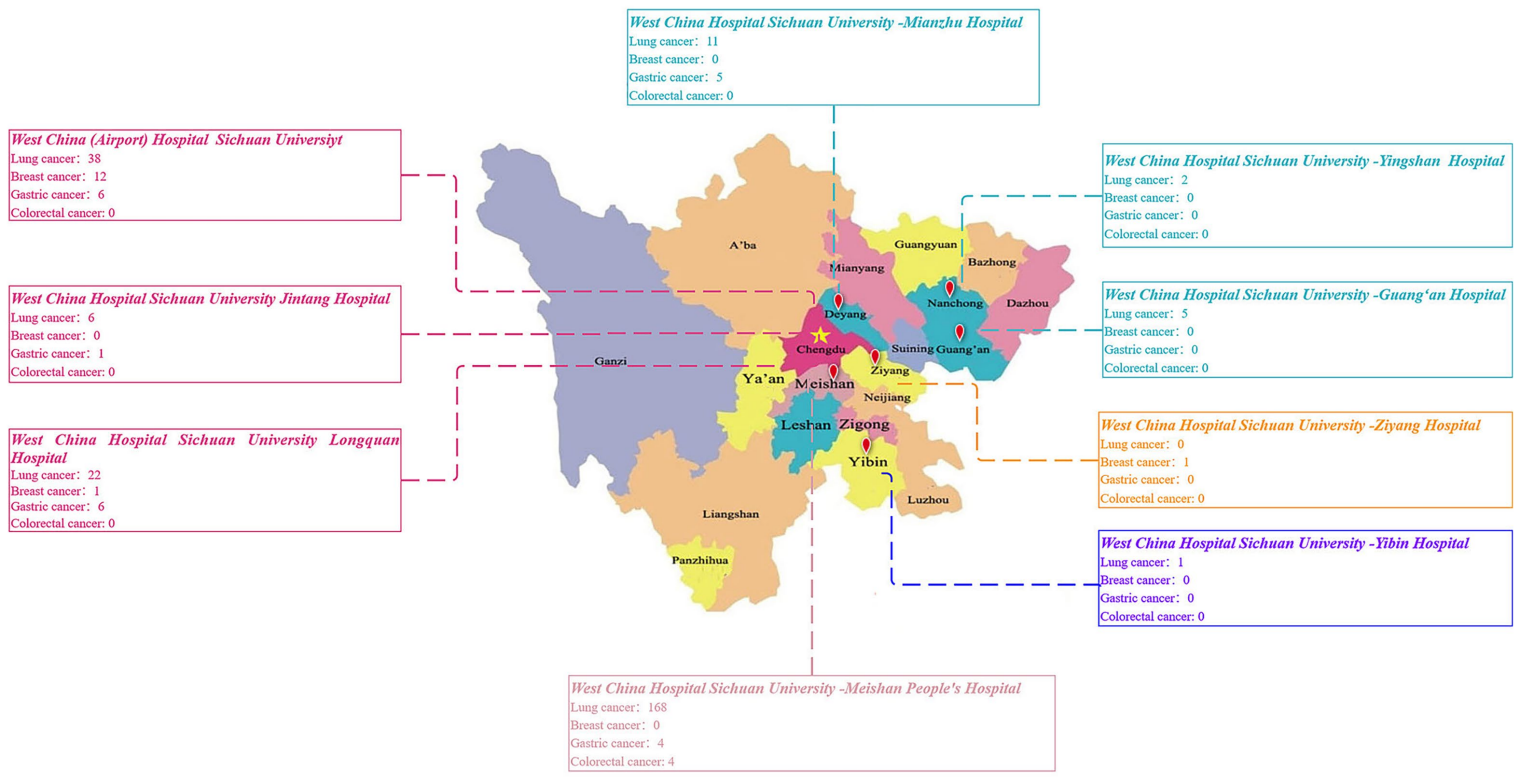
Distribution of patients enrolled in disease-specific programs across the medical alliance.

As of July 2025, the platform had enrolled 293 patients across the four disease-specific pathways, including 253 with lung cancer, 14 with breast cancer, 22 with gastric cancer, and 4 with colorectal cancer. The regional distribution of patient enrollment is presented in Figure 6. Taken together, these findings demonstrate the platform’s capacity for multicenter recruitment of diverse patient populations, providing a robust foundation for subsequent multidimensional clinical and translational research.

### 3.2 Exemplary results for the lung cancer disease-specific pathway

At present, the lung cancer pathway at Meishan People’s Hospital represents the most mature application of the W-SDM System. By integrating with the central hospital’s disease-specific database, a multicenter and heterogeneous research cohort has been successfully established. The baseline data distribution is summarized in Table 2, with clinical staging information being comprehensive and reliable for the majority of enrolled patients. The cohort encompasses both early- and late-stage cases, and its wide geographic coverage further indicates that the W-SDM System effectively supports the enrollment of heterogeneous patient populations. These features ensure sample diversity while maintaining data completeness and consistency across multiple centers.

**Table 2:** Baseline characteristics of patients with lung cancer (*n* = 168).

Characteristic	Lung cancer (*n* = 168)
Age, years	—
Mean ± SD	63.03 ± 11.49
Median (IQR)	63.00 (56.00–71.00)
Sex, *n* (%)	
Male	54.59
Female	45.41
Smoking history, *n* (%)	
Current smoker	11.02
Former smoker	10.23
Never smoker	27.5
Comorbidities, *n* (%)	
Hypertension	20.69
Diabetes	6.32
COPD	8.46
TNM stage, *n* (%)	
Stage 0	0.79
Stage IA1	16.54
Stage IA2	28.35
Stage IA3	2.36
Stage IB	14.96
Stage IIA	3.15
Stage IIB	2.36
Stage IIIA	3.15
Stage IIIB	0.79
Stage IIIC	0.79
Stage IVA	16.54
Stage IVB	0.79
Occult cancer	0.79
NA	8.66
New/returning, *n* (%)	
First visit	83.91
Follow-up visit	16.09

### 3.3 Data management

To ensure the compliant utilization of multicenter data within the disease-specific medical alliance system, the central hospital and participating member institutions signed a Data Integration and Management Agreement. In alignment with relevant national laws and regulations—including the Personal Information Protection Law, the Data Security Law, and the Administrative Measures for Cybersecurity in Medical and Health Institutions of the People’s Republic of China—the agreement defines the respective responsibilities and rights regarding data sharing. It underscores the adoption of de-identification and anonymization measures during data processing to safeguard patient privacy and information security. Under this governance framework, member hospitals are able to integrate and share clinical data based on unified standards, thereby supporting the redistribution of high-quality medical resources and enabling collaborative research. Ultimately, this establishes a standardized and secure data infrastructure to support subsequent multicenter clinical and translational studies.

### 3.4 Quantitative verification of multicenter data integration quality

As of July 2025, the project completed its initial round of data pathway verification and quality spot checks to assess the integrity, consistency, and interoperability of multicenter data integration. A multidimensional evaluation framework was constructed to assess data governance performance across medical alliance institutions. The framework comprised five governance domains: Structured Data Representation Integrity (maximum 5.5 points), Data Quality Compliance (maximum 1.5 points), Governance Agreement Execution (maximum 2.0 points), Connectivity Performance (maximum 3.5 points), and Backup Repository Submission (maximum 1.5 points). All domains were operationalized using predefined binary validation rules, based on the structured data verification procedures described previously. Structured Data Representation Integrity was evaluated according to the completeness of routine monthly reporting and the development progress of 71 standardized structured interface tables. Data Quality Compliance was assessed through structural completeness and logical consistency validation of clinical, imaging, and laboratory datasets. Governance Agreement Execution reflected the formal implementation of institutional data-sharing agreements. Connectivity Performance was determined by cross-institutional interface debugging and data transmission testing results. Backup Repository Submission was evaluated based on verified confirmation of secondary data repository transfer.

The scoring process was conducted by the central hospital’s information management team, and the results were formally reviewed and signed off by each participating alliance institution, thereby establishing a structured and accountable data quality governance mechanism within the medical alliance. Domain scores were aggregated according to predefined weighting coefficients to generate composite institutional scores (see Supplementary Table), enabling standardized cross-institutional comparison within the medical alliance. The institutions were ranked according to their composite scores. Meishan People’s Hospital achieved the highest score (16.00/16.00), followed by Shuangliu District First People’s Hospital (14.64/16.00) and Longquanyi District First People’s Hospital (14.33/16.00). Institutions with lower composite scores were primarily limited by either reduced case contribution volume or incomplete backup repository submission compliance.

### 3.5 Informed consent to promote pathway standardization and research compliance

To strengthen patient management and ensure standardized implementation of clinical pathways, all patients in the disease-specific alliance hospitals provided written informed consent prior to enrollment, which was securely uploaded into the system. The consent process was designed to be patient-centered, with clear communication of the objectives, procedures, and potential risks of the clinical pathway, thereby safeguarding patient autonomy and informed decision-making. From a clinical management perspective, the consent process not only enforces adherence to evidence-based and standardized pathways but also establishes a compliant, high-quality data source and governance framework to support multicenter clinical research and medical education.

## 4 Discussion

### 4.1 Principal findings

This study demonstrates the feasibility and practical value of developing and implementing the W-SDM System within a medical alliance setting. By coordinating mobile and PC interfaces, the platform enables disease-specific physicians across different hospitals to overcome geographical barriers, supporting real-time monitoring of clinical work and pathway execution. At the same time, it provides guidance experts with tools for multicenter data collection and pathway standardization, supporting cross-institutional, standardized clinical workflows and coordinated data management.

System development required integration with core hospital systems and accommodation of multidisciplinary team requirements. To address these challenges, a hybrid development strategy combining prototyping and rapid application development (RAD) was employed, allowing rapid capture and iteration of requirements while ensuring efficient modular development and parallel testing. This approach effectively managed the complexity of multi-campus, cross-team, and heterogeneous requirements under tight timelines, ensuring timely platform deployment and accommodating the customized needs of various disease-specific teams, serving as a replicable model for patient management platforms in medical alliances.

The unified data interconnection architecture and structured clinical pathway forms were critical to system success. Standardized data structures, secure data transmission mechanisms, and multicenter interfaces support traceable and interoperable data flow across alliance hospitals. These architectural features provide the structural foundation for pathway comparison, deviation identification, and future data-driven optimization.

Interface design across mobile and PC platforms was guided by clinical workflow habits. The structured representation of clinical pathways facilitates core functionalities. The layered mobile–PC configuration supports data entry, pathway visualization, and quality supervision across user groups, forming an operationally coherent framework for alliance-level pathway governance. By providing structured pathway visualization and deviation alerts, the system establishes process-level transparency that may support improved transparency in pathway execution and facilitate pathway governance across the alliance.

To better contextualize this study, we compared the W-SDM System with existing digital health platforms and integrated care models. Prior studies have shown that electronic health record (EHR) systems can support standardized clinical processes and data-driven coordination; however, variability in digital maturity across institutions may limit interoperability and effective collaboration, particularly in the absence of unified data standards (30). The World Health Organization has emphasized that integrated care relies on interoperable digital infrastructures to enable continuous and coordinated service delivery across different levels of care (31). In addition, evidence suggests that structured clinical pathways can improve clinical practice and promote standardized care delivery (32). In this context, the W-SDM System establishes a pathway-centered structured digital framework with unified data standards, providing a feasible approach to standardized management and cross-institutional collaboration in heterogeneous multi-center environments.

### 4.2 Limitations and future work

Several challenges remain. Although 11 hospitals participated in the study, 60.1% (176/293) of enrolled patients were from Meishan People’s Hospital. This imbalance likely reflects variations in clinical and informatics capabilities among alliance hospitals, with heterogeneity in data processing and workflow standardization affecting system adoption and operational consistency. Additionally, differing optimization needs and usage habits across disease-specific teams impose higher demands on ongoing training and iterative system refinement. Usage statistics indicated that in 2024, the central hospital’s information center deployed dedicated personnel and conducted monthly training sessions for alliance hospitals; however, in 2025, this initiative was not maintained, resulting in a 45% month-on-month decrease in patient enrollment in January 2025. These findings highlight the critical importance of continuous advocacy, training, and cross-institutional communication for effective platform utilization and research progression. Furthermore, as patient numbers increase, manual pathway quality checks become increasingly labor-intensive. Future work will explore the application of artificial intelligence to compare structured clinical pathway data against standardized pathways, automatically generating reference-quality control outputs to reduce manual workload.

It should be noted that improving clinical consistency and optimizing operational coordination are among the key objectives for the W-SDM System in its later-stage implementation. Although these aspects could not be quantitatively assessed in the present early-stage study, we anticipate that the system will progressively contribute to these goals as it is further deployed and applied. Future studies will incorporate longitudinal follow-up and controlled analyses to more rigorously evaluate the system’s potential impact in these areas.

Finally, this study represents an early implementation phase. The sample size and follow-up duration remain limited. Future research will incorporate longitudinal follow-up and controlled evaluations to more rigorously assess clinical outcomes, pathway adherence, and patient-level impact.

## 5 Conclusion

This study demonstrates that the W-SDM System provides a feasible and scalable digital infrastructure for structured clinical pathway governance within a medical alliance. By integrating unified data interconnection architecture, standardized pathway forms, and coordinated mobile–PC interfaces, the platform enables cross-institutional collaboration, real-time pathway supervision, and traceable data flow across heterogeneous hospital environments. The implementation experience highlights that beyond technical architecture, sustained institutional engagement, training support, and governance coordination are critical determinants of system effectiveness. Structured and interoperable pathway data create the necessary foundation for deviation visualization, multicenter comparison, and future data-driven optimization. Although the current evaluation remains preliminary, the system establishes an operational framework for alliance-level pathway management and provides a potential replicable model for digital patient management platforms in multi-hospital settings. Future integration of automated quality control and longitudinal outcome evaluation will further strengthen its clinical and research value.

## Data Availability

The raw data supporting the conclusions of this article will be made available by the authors, without undue reservation.
