# Supplementary material for: Multi-center disease-specific management system: toward standardized and harmonized clinical pathways in integrated care networks
Source: Front Public Health. 2026 May 7;14:1764254. doi: 10.3389/fpubh.2026.1764254 (PMC13190477; doi:10.3389/fpubh.2026.1764254)
Supplement: Supplementary file 1 [file Data_Sheet_1.zip › Supplementary materials/Supplementary_Tables.docx]

| ***Medical Alliance Data Integration and Consistency Validation Entries*** | | | |
| --- | --- | --- | --- |
| **Data Validation Type** | **Data Validation Content** | **Data Validation Description** | **Validation Method Description** |
| Data Integrity | Patient Baseline Data Integrity | Determine the total number of actual hospital admissions to provide a baseline for all subsequent data validations. | Purpose: Establish a reference for completeness assessment.  Method: Count total records in the inpatient patient index table (T_Inpath_Patients). This value serves as a baseline for subsequent consistency checks and coverage analysis.. |
|  | Outpatient/Inpatient Settlement Integrity | Verify completeness of billing linkages across patient visit → patient index → ID number → inpatient patient master table. | Purpose: Ensure full linkage of billing and patient records.  Method: Stepwise SQL query: extract patient type, visit serial number, and settlement ID from central billing table (TB_ZX_JSMX); link to inpatient (TB_ZY_RYDJMXB) or outpatient (TB_MZ_JZMXB) tables; obtain patient main index; link to patient info table (TB_HZXX) to get ID number; verify presence in T_Inpath_Patients |
| Data Consistency | Patient Identity Consistency Validation | Match ID numbers between patient info table and inpatient patient table; check unique visit-patient combinations. | Purpose: Ensure identity consistency and data integrity.  Method: Extract visit serial number and visit number from TB_HZXX; link with T_Inpath_Patients via ID number; deduplicated count reflects matching of patient info and registration data. |
|  | Inpatient Medication Record Consistency Validation | Verify inpatient identity and consistency between admission and medication records. | Purpose: Confirm medication records belong to standard inpatient pool. Method: Extract unique inpatient visit numbers from TB_ZY_RYDJMXB; link with TB_HZXX and T_Inpath_Patients; cross-check with inpatient medication table (TB_CDA_ZYYPYZMX); count successfully matched record. |
|  | Inpatient Surgery Record Consistency Validation | Validate patient identity in surgical records against standard inpatient database. | Purpose: Ensure all surgery records correspond to known inpatients.  Method: Extract surgical IDs (SSID) and patient main index (GRBSLX, GRBSH) from TB_SS_SSJLB; link patient main index to TB_HZXX to retrieve ID number; match with T_Inpath_Patients. |
| Cross-System Consistency | Prescription Data Association Validation | Verify consistency of patient identity across outpatient prescription, visit, and inpatient systems. | Purpose: Ensure prescription records map correctly to inpatient patients.  Method: Extract unique prescription numbers and visit numbers from TB_CDA_MZYPCF; link to outpatient visit table (TB_MZ_JZMXB) to get patient main index; connect with TB_HZXX to obtain ID number; compare with T_Inpath_Patients; aggregate matched records for consistency evaluation. |
| Data Fusion Quality | Cross-Database Inpatient Patient Information Consistency Validation | Confirm inpatient info consistency with standard patient database. | Purpose: Ensure accuracy and integrity of inpatient identity.  Method: Extract inpatient visit numbers from TB_ZY_RYDJMXB; obtain patient main index; link with TB_HZXX to retrieve ID number; match with T_Inpath_Patients. |

| ***Structured Pathway Form and Data Feedback for Lung Cancer*** | | | | | | | |
| --- | --- | --- | --- | --- | --- | --- | --- |
| **Disease Entity** | **Classification Standard** | **Visit Stage** | **Staging Basis** | **Clinical Domain** | **Clinical Pathway Indicator** | **Indicator Definition** | **Structured Clinical Data for Feedback** |
| Pulmonary Nodule | In Accordance with ICD-10 Coding System (Pulmonary Nodule). Selected by doctor upon patient entry. | First Visit (1st time) | Determined by department; if not met, considered unscheduled. | Lung Cancer Screening | Age Group | Recommended starting age for lung cancer screening is 40. | 1) Age at visit |
|  |  | Recheck (2nd time) |  |  | Medical History | High-risk history, such as smoking, family history, occupational exposure, etc. | 1) Electronic Medical Record – Admission Note – Past Medical History； 2) Family History； 3) Personal History (including life experience, residential and travel history, occupation and working conditions, lifestyle and habits, history of recreational drug use, and exposure to medications, foods, and other allergens) |
|  |  | Recheck (3rd time) ... |  |  | Imaging Exam | LDCT recommended for pulmonary nodule detection. | 1) CT Report – Imaging Findings； 2) CT Report – Impression |
| Lung Cancer | In Accordance with ICD-10 Coding System (Lung Cancer). Selected by doctor upon patient entry. | First Visit (1st time) | Determined by department; if not met, considered unscheduled. | Basic Info | Diagnosis | Outpatient and inpatient diagnosis | Outpatient/inpatient record |
|  |  |  |  |  | Clinical Presentation | Symptoms caused by local growth of primary lung tumor: cough, hemoptysis, dyspnea, fever, wheezing | 1)EMR - Admission note: chief complaint;  2)history of present illness; 3)physical examination |
|  |  |  |  | Examination | Imaging | Diagnosis: LDCT recommended; Staging: 3D reconstruction of pulmonary vessels (CT) | Imaging report |
|  |  |  |  |  | Laboratory Tests | Sputum cytology, serum lung cancer markers, bronchoalveolar lavage markers, 7 lung cancer autoantibodies, CTCs, ctDNA, DNA methylation, exhaled breath analysis | Laboratory report |
|  |  | Recheck (2nd time) |  |  | Bronchoscopy | - | Bronchoscopy report |
|  |  |  |  |  | Histopathology | Immunohistochemistry and special staining | Pathology report |
|  |  |  |  |  | Molecular Pathology | Targeted gene testing, immunotherapy-related biomarkers (PD-L1, TMB) | Genetic test report |
|  |  |  |  | TNM Staging | TNM | TNM staging results | TNM report |
|  |  |  |  | Treatment | Surgery | Thoracoscopic/minimally invasive surgery recommended, open surgery as supplement | Surgical record |
|  |  |  |  |  | Radiotherapy | Principles of lung cancer radiotherapy, NSCLC and limited-stage SCLC radiotherapy | Orders - Radiotherapy - Dose |
|  |  |  |  |  | Chemotherapy | TP/TC, nab-TP/TC, AP/AC regimens | Orders - Chemotherapy - Drugs |
|  |  | Recheck (3rd time) ... |  |  | Targeted Therapy | TKIs, monoclonal antibodies, multi-target drugs | Orders - Targeted therapy - Drugs |
|  |  |  |  |  | Immunotherapy | NSCLC, Small Cell Lung Cancer (SCLC) | Orders - Immunotherapy - Drugs |
|  |  |  |  |  | Interventional Therapy | Includes percutaneous, airway, and vascular routes; covers both local tumor therapy and complication management | Orders - Interventional therapy - Procedure name |
|  |  |  |  |  | Biological Therapy | CRISPR/Cas9-modified T cells and personalized dendritic cell vaccines | Orders - Biological therapy - Drug name |

| ***Multidimensional Evaluation of Medical Alliance Institutional Data Governance Performance*** | | | | | | | | | | | | | |
| --- | --- | --- | --- | --- | --- | --- | --- | --- | --- | --- | --- | --- | --- |
| **Institution** | **Total Cases** | **Lung Cancer** | **Gastric Cancer** | **Colorectal Cancer** | **Breast Cancer** | **Data Pipeline Integrity (5.5)** | **Data Quality Compliance (1.5)** | **Governance Agreement (2.0)** | **Data Connectivity Performance (3.5)** | **Backup Repository Submission (1.5)** | **Governance Capacity Score (14)** | **Case Contribution Score (2)** | **Composite Score (16)** |
| Meishan People’s Hospital | 176 | 168 | 4 | 4 | 0 | 5.5 | 1.5 | 2 | 3.5 | 1.5 | **14** | 2 | **16** |
| Shuangliu (Airport)People’s Hospital | 56 | 38 | 6 | 0 | 12 | 5.5 | 1.5 | 2 | 3.5 | 1.5 | **14** | 0.64 | **14.64** |
| Longquanyi District First People’s Hospital | 29 | 22 | 6 | 0 | 1 | 5.5 | 1.5 | 2 | 3.5 | 1.5 | **14** | 0.33 | **14.33** |
| Mianzhu People’s Hospital | 16 | 11 | 5 | 0 | 0 | 5.5 | 1.5 | 2 | 3.5 | 1.5 | **14** | 0.18 | **14.18** |
| Jintang County First People’s Hospital | 7 | 6 | 1 | 0 | 0 | 5.5 | 1.5 | 2 | 3.5 | 1.5 | **14** | 0.08 | **14.08** |
| Guang’an People’s Hospital | 5 | 5 | 0 | 0 | 0 | 5.5 | 1.5 | 2 | 3.5 | 1.5 | **14** | 0.06 | **14.06** |
| Ziyang Central Hospital | 1 | 0 | 0 | 0 | 1 | 5.5 | 1.5 | 2 | 3.5 | 1.5 | **14** | 0.01 | **14.01** |
| Yibin Second People’s Hospital | 1 | 1 | 0 | 0 | 0 | 5.5 | 1.5 | 2 | 3.5 | 1.5 | **14** | 0.01 | **14.01** |
| YingShan Hospital | 2 | 2 | 0 | 0 | 0 | 5.5 | 1.5 | 2 | 3.5 | 0 | **12.5** | 0.02 | **12.52** |

**Governance Capacity Score (maximum 14.0 points)** was derived from five predefined governance domains;**Data Pipeline Integrity (maximum 5.5 points)** was determined based on the completeness of routine data reporting and the development status of 71 standardized structured interface tables;**Data Quality Compliance (maximum 1.5 points)** reflected structural completeness and logical consistency validation across clinical (52 tables), imaging (11 tables), and laboratory (8 tables) datasets.**Governance Agreement Execution (maximum 2.0 points**) indicated the formal completion and implementation of institutional data-sharing agreements**;Connectivity Performance (maximum 3.5 points)** represented successful cross-institutional interface debugging and verified data transmission testing.**Backup Repository Submission (maximum 1.5 points)** indicated confirmed secondary data repository transfer and archival verification;**The Case Contribution Score (maximum 2.0 points)** was calculated using a normalized Case Contribution Index (CCI), defined as:

CCI = (Institution Valid Case Count / Maximum Case Count within the Alliance) × 2.0

**The Composite Score (maximum 16.0 points)** represents the sum of Governance Capacity Score and Case Contribution Score. Institutions were ranked in descending order according to their composite scores.
