# Supplementary figures and images for: Multi-center disease-specific management system: toward standardized and harmonized clinical pathways in integrated care networks
Source: Front Public Health. 2026 May 7;14:1764254. doi: 10.3389/fpubh.2026.1764254 (PMC13190477; doi:10.3389/fpubh.2026.1764254)

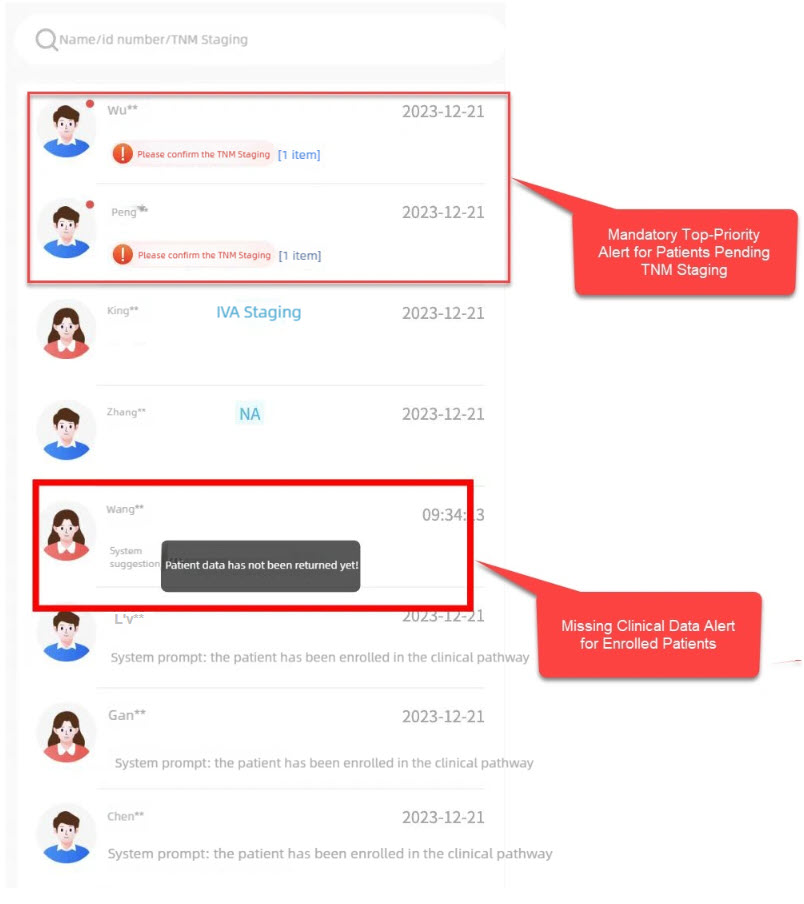

Supplement: Supplementary file 1 [file Data_Sheet_1.zip › Supplementary materials/Supplementary Figure S3.jpg]

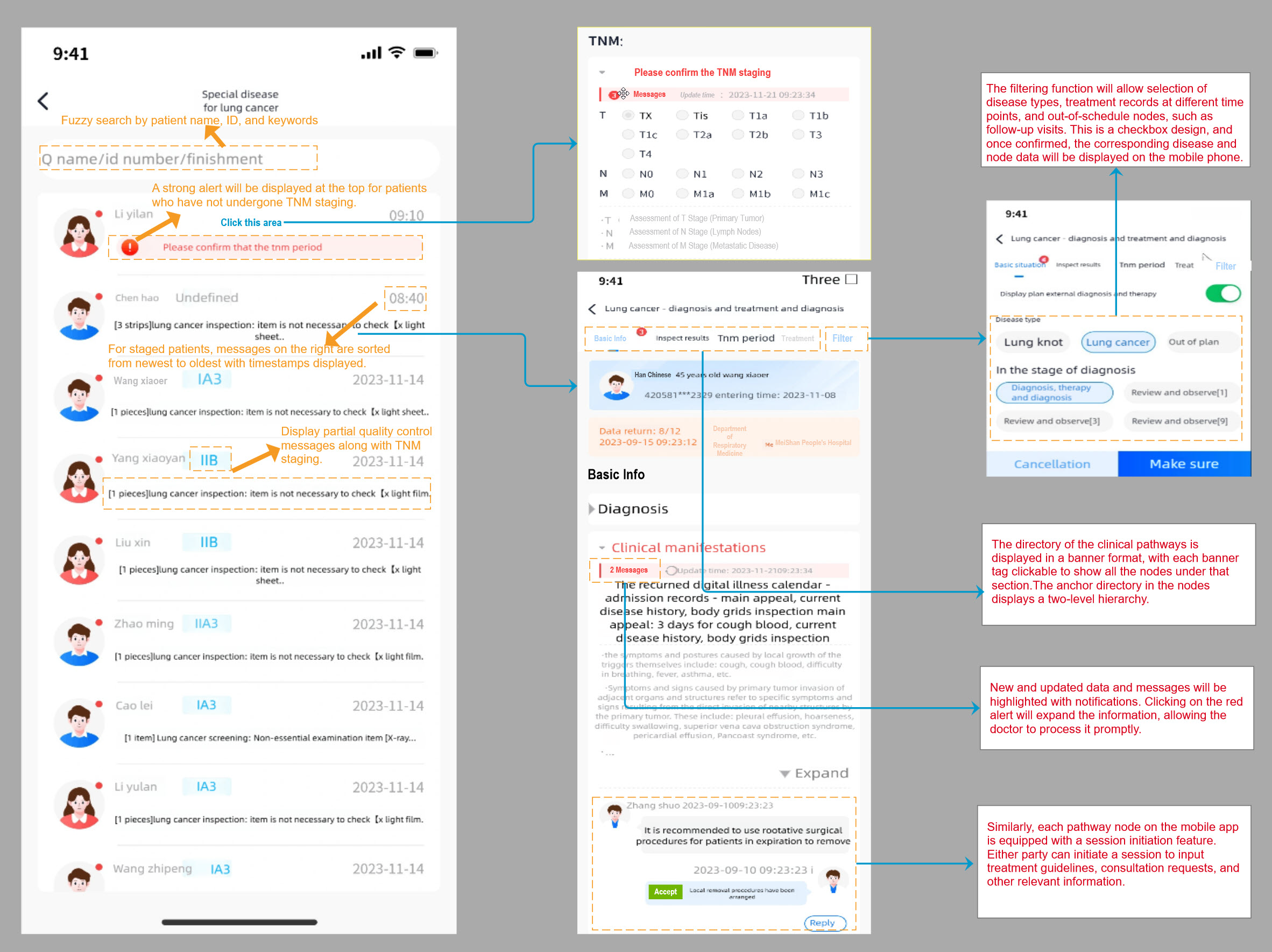

Supplement: Supplementary file 1 [file Data_Sheet_1.zip › Supplementary materials/Supplementary Figure S1.png]

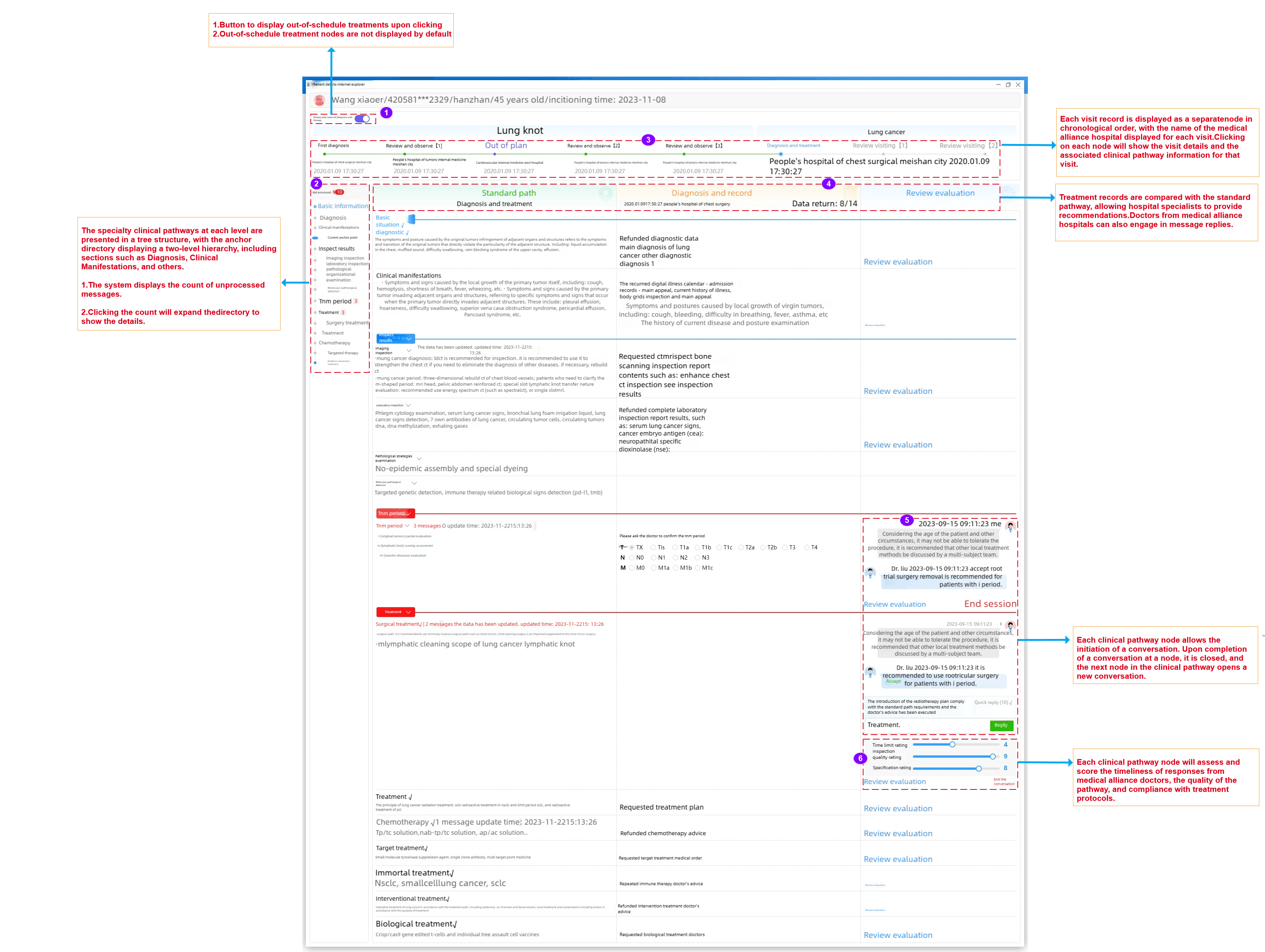

Supplement: Supplementary file 1 [file Data_Sheet_1.zip › Supplementary materials/Supplementary Figure S2.png]

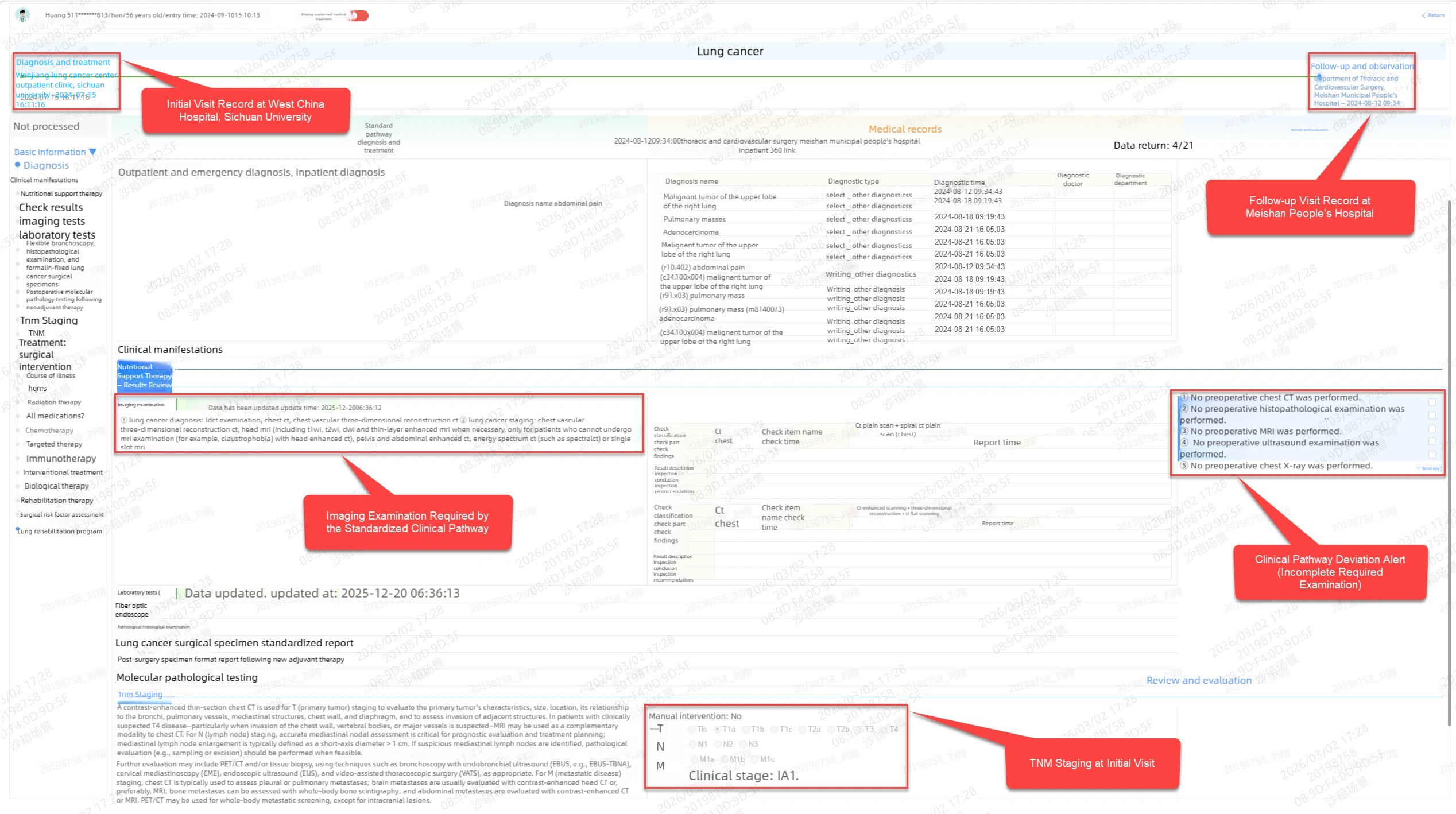

Supplement: Supplementary file 1 [file Data_Sheet_1.zip › Supplementary materials/Supplementary Figure S4.png]
